# Differentiating Identities Within an Extreme Sport: A Case Study of Mountain Biking Print Advertisements

**DOI:** 10.3389/fpsyg.2018.01668

**Published:** 2018-09-26

**Authors:** Kieren McEwan, Neil Weston, Paul Gorczynski

**Affiliations:** Department of Sport and Exercise Science, University of Portsmouth, Portsmouth, United Kingdom

**Keywords:** mountain biking, identity, risk-taking, competition, extreme sport

## Abstract

The work of McEwan (2016) has questioned the assumed homogeneity of mountain biking in terms of culture and sporting values, leading to the suggestion that there may be differing patterns of identity within the various formats of the sport. This is also supported by McEwan and Weston (2017) findings, which advanced knowledge of the mountain biking industry by defining the differing pluralized segments within the market and highlighting their hierarchical nature in relation to the cost of products. This therefore leads to a question over whether differing markets are reflected in differing identities among varying consumer groups within the sport of mountain biking. Thus, this study sought to establish what these identity characteristics are through an analysis of a sample of mountain biking magazine advertisements (*N = 568*). The analysis was conducted using a sequential Ethnographic Content Analysis (Altheide, 1987) followed by a Quantitative Content Analysis (Berelson, 1952) mirroring the approaches used by Williams et al. (2010) and Cann (2012) in their studies of the portrayal of identity within magazine advertisements. Results of this analysis highlighted five identity characteristics (places of play, equipment functionality, risk taking, competitiveness, activity aesthetics), three of which varied significantly between differing formats of mountain biking (risk taking, competitiveness, activity aesthetics). Activity aesthetic was established as a component of risk-taking rather than an independent identity characteristic and therefore risk taking along with competitiveness formed the basis of a refined four-format activity categorization within mountain biking and the production of a model of participant archetypes.

## Introduction

The collective activities that would become known as extreme sports, of which mountain biking is one example, emerged as a reaction against social living patterns (Donnelly, 1988) and developed an ethos that set them apart from mainstream sports culture (Maguire, 1999). They have been described as lifestyle sports (Wheaton, 2004a) with strong subcultural values (Wheaton and Beal, 2003; Wheaton, 2007; McEwan, 2016) and associations to patterns of consumerist consumption (Wheaton, 2013). Such patterns of consumption impact upon the identities present within extreme sports and this study sought to address this point through an analysis of the sport of mountain biking.

Since its birth, mountain biking has evolved pluralistically into a complex arrangement of specialist activities (McEwan and Weston, 2017), which have been suggested to present differing subcultural values (McEwan, 2016). However, this remains largely unexplored within the literature and raises questions over the homogeneity of mountain biking as a unified culture. McEwan and Weston (2017) demonstrated how the mountain bike market consists of six market segments, each offering products designed for differing types of riding and thus facilitating particular behaviors. In postmodernity, products allow individuals to demonstrate self-symbolism (Elliott, 1997) and advertisements for consumables serve to create and reinforce identity (Elliott and Wattanasuwan, 1998; Elliott and Davies, 2006). This study sought to bring these concepts together in an investigation of identities depicted within mountain bike magazine advertisements.

Identity itself is a reflexive characteristic (Goffman, 1959) and the fluid nature of ‘self’ represents a constructed face that individuals present within social interactions (Gergen, 1965, 1982; Turner, 1988). As a “reflexive project,” (Giddens, 1991, p.32) identity is constructed, selected and ultimately managed as a process of self-representation. Individuals select how they want to represent themselves and be seen by the outside world. Equally social identity plays a constructive role in the facilitation and formation of relationships (Tajfel, 1972, 1978) and therefore the groups that individuals are associated with play a component part in defining their identity. Identity can facilitate membership of a group but the desire to enter social configurations can influence individuals to alter or adapt their identity in order to achieve association. This can clearly be viewed within extreme sports through the styles of dress, language, etiquette and even subcultural values adopted by participants (Beal, 1995, 1996, 1998; Anderson, 1999; Heino, 2000; Beal and Weidman, 2003; Wheaton and Beal, 2003; Wheaton, 2003, 2004a,b, 2007, 2010; Bennett and Lachowetz, 2004; Donnelly, 2006; Rinehart, 2007; McEwan, 2016).

Research on extreme sports has seized upon the attachment of these ‘new’ sports to cultural and identity narratives, which are at odds with mainstream sports culture (Midol, 1993; Beal, 1995, 1996; Le Breton, 2000; Wheaton, 2003, 2004a,b, 2007, 2010, 2013; Wheaton and Beal, 2003; Bennett and Lachowetz, 2004; Puchan, 2005; Donnelly, 2006). The identities assumed by those taking part in these emerging sports is important as it allows participants to define themselves as subcultural devotees through a demonstration of authenticity (Beal and Weidman, 2003; Wheaton and Beal, 2003). In order to conform, the serious participant must opt into the group identity in order to establish membership. Cultural characteristics therefore become tangible as a reflection of subcultural values and embody the sport’s identity narratives (Heino, 2000; Donnelly, 2006) and in particular their masculinised nature (Anderson, 1999; Sisjord, 2009; Thorpe, 2010; Huybers-Withers and Livingston, 2010; Huybers-Withers, 2015). The question here, therefore, centers on the how identities are depicted and promoted to participants through advertising materials related to mountain biking products.

McEwan and Weston (2017) formally observed the pluralized nature of mountain biking and drew attention to the need for further analysis of the different identities within the sport in order to understand how differing participant narratives present themselves. This study sought to explore this with the objective of establishing core idealized identity narratives within mountain biking and defining how they might differ between the various formats of the sport. The need to carry out such research stems from a desire to establish how mountain bike markets marry up to participant identity narratives, thus developing practicable knowledge for those marketing products and services to consumers. In this respect advertisements play a significant role in the portrayal of identity to target markets (see Consterdine, 2002; Williams et al., 2010; Cann, 2012). This is particularly important, as it has been shown that consumers gravitate toward products, services and brands that reflect identities that they possess (Bhattacharjee et al., 2014).

The bicycle market has tended to be viewed as a flourishing industry over recent years. As an example of this, sales of bicycles in the United Kingdom alone rose by 14% between 2008 and 2013 (Mintel, 2014) and this is representative of a trend that has been replicated globally (see Confederation of the European Bicycle Industry, 2015). This has also led to large increases in profits for multinational cycle manufacturers, such as Shimano (Bicycle Retailer, 2016). However, reports within the cycling industry since 2015 have begun to focus on stagnation with the cycling market (see Harker, 2015; Sutton, 2015; Reid, 2018) and this represents a significant challenge to those marketing products within an increasingly challenging consumer landscape. A well-established solution to stagnating markets is to focus on clear strategies to cultivate interest amongst consumers (Estelami, 2010). One way of doing this is by better understanding consumer groups (Ames and Hlavacek, 1984) and thus being in a more advantageous position to market products more effectively. Therefore, an analysis of identity in mountain biking, as a core element of the cycling industry, will be of major benefit to those marketing products to consumers.

Therefore, the study sought to achieve three particular research aims: firstly, to establish the identity characteristics within the sport of mountain biking and secondly, to establish and map how these identities differ between various formats of the sport. As a third and final aim, the ambition was to utilize the knowledge gained while addressing aims 1 and 2 in order to develop a model of mountain biker identity that could form the basis of future research.

## Materials and Methods

The study was split into three phases. In the initial inductive phase, an Ethnographic Content Analysis (ECA; Altheide, 1987) was conducted, which allowed key identity characteristics to emerge through thematic semiotic analysis using the same approach used by both Williams et al. (2010) and Cann (2012). In phase two, a Quantitative Content Analysis (QCA; Berelson, 1952) was employed to establish if there were any identity differences evident between the various forms of mountain biking. These first two phases fundamentally conform to the qualitative-quantitative sequential triangulation design described by Creswell (2003). The third phase of the study consisted of a secondary analysis of the QCA data using a Multidimensional Scale Analysis (MDS; Kruskal and Wish, 1978). This echoed the approach used by McEwan and Weston (2017) and made it possible to visually map the proximal similarities and differences of the various formats of mountain biking.

### Materials

The study made use of the advertisements taken from four United Kingdom based mountain bike magazine publications (*Mountain Bike Rider*, *Dirt Mountain Bike Magazine*, *Mountain Bike UK*, & *What Mountain Bike*). It is noted that other sources could have been used (e.g., websites), however magazines remain a prominent marketing communication channel and it was felt that studying advertisements placed by manufacturers to engage with consumers in the print media would give a valid insight into idealized mountain bike identity. Each magazine was published on a monthly basis with large circulation rates and the sample was drawn from two years (2009 and 2013) to ensure stability within the data. Advertisements that appeared multiple times, (either in different magazines or repeated in various editions of the same publication) were included only once within the sample.

Only advertisements that could clearly be attached to particular formats of mountain biking were included within the study. However, it was noted that several formats identified by McEwan and Weston (2017) were either not represented within the sample (trials riding and marathon racing) or were present only in low numbers (slope style *n* = 4, northshore riding, *n* = 7 and four-cross racing *n* = 9). These formats represented only 3.4% of the original sample of 588 advertisements and in order to avoid drawing incorrect conclusions from such a small number of advertisements related to these formats, they were withdrawn from the study. The remaining sample of 568 advertisements was made up of eight categories of mountain biking, consisting of cross country (*n* = 77, raced on relatively flat courses), downhill and enduro downhill (*n* = 106 and *n* = 27, raced on short or long downhill courses, respectively), trail riding (*n* = 142, recreational trail riding in the countryside), all mountain (*n* = 71, recreational trail riding in the high mountains), freeriding (*n* = 73, off-piste riding often in the high mountains), dirt jumping and street riding (*n* = 48 and *n* = 24, trick orientated mountain biking).

## Phase 1: Ethnographic Content Analysis (ECA) Method

In order to evaluate and classify the advertisements, an ECA was conducted with a focus on the semiotic appraisal of the sample material (see Krippendorff, 1980; Schroeder, 2006). Content analysis methodologies have been traditionally quantitative in nature (e.g., Neuendorf, 2002), however the use of this approach within a qualitative paradigm has become increasing common (see Mayring, 2004) and the study design used within the first phase of this research utilized the approach developed by Altheide (1987). The aim within the first phase of the study was to discern the identities portrayed within the sample as a whole and draw out the cultural meanings in relation to the various formats of mountain biking. In this sense, the initial phase of the study sought to establish the subcultural values across all forms of mountain biking.

Ensuring the validity of the observations made in the ECA phase of this study required consideration. As Daymon and Holloway (2011) point out, to analyze advertisements using semiotics requires the researcher to explore the cultural communication between the seller and the consumer. However, such communication is aimed at an informed consumer market, and therefore cultural messages often cannot be easily perceived by those unfamiliar with the culture itself.

The primary researcher initially established codes and identity themes through interpretive observation. In each individual case the advertisements were observed and evaluated through a semiotic process (see Eco, 1976) based on their content. To this extent, both the imagery and text present within each individual advertisement was evaluated and deciphered to discern an observable cultural meaning. These initial observations led to a large number of lines of content. The lead researcher then took these and inductively analyzed them to form codes, by drawing together and grouping observations based on the raw material generated within the sample. The descriptions shown within the tables in the results section below represents a summary of the lines of content that made up each individual code.

Given the large sample used within this study and the array of observations made, the semiotic approach used became key within the process. Once the codes were established these were then evaluated and drawn into higher order identity themes. During this phase of analysis, a focus was also placed on separating identity themes, which were core within mountain biking as a whole from those that appeared to be represented more frequently in particular formats. These differing identity themes were then carried forward into the analysis conducted within the second phase of this study.

Ordinarily good practice would be to conduct thematic coding and data reduction that is independently verified by fellow researchers. However, the obvious insider culture present within extreme sports, such as mountain biking meant that an additional validation process was needed within this study. In this case the primary researcher was an active mountain biker with over eighteen years of experience within the sport, who therefore possessed an insider knowledge of the culture along with expertise in content analysis methods. Two researchers also with experience of using content analysis methodologies corroborated the established themes, but both possessed a limited knowledge of mountain bike culture. In order to mitigate against this, themes were additionally verified by two experienced mountain bikers, both with over fifteen years of experience within the sport. Using this approach meant that the codes developed within this study were verified methodologically (by fellow researchers) and culturally (by mountain bikers), making the semiotic process more reliable.

The codes themselves were developed inductively as there was no preconceived idea of the content that would be portrayed within the advertisements. These codes were then clustered in identity themes through the semiotic process at the heart of ECA (Altheide, 1987) and reflecting the process used by Williams et al. (2010) and Cann (2012).

## Phase 1: ECA Results

The ECA results established 11 codes that were arranged under five identity themes within mountain biking (see **Tables 1**, **2**). These included: the sites where mountain biking takes place (places of play); the use of complex and high-tech equipment (equipment functionality); the attachment to traditional forms of competition (sporting characteristics); the association of mountain biking to danger and risk taking (the importance of risk); and finally artistic creativity (activity aesthetics).

**Table 1:** The two core identity characteristic themes associated with all mountain biking.

Identity themes	Codes	Description of observations
Places of play	Man-made spaces	The use of non-natural resource spaces fell into two categories. These constituted the use of specifically designed spaces for mountain biking (e.g., jumps built specifically for freestyle forms of mountain biking) or where riders used the cityscape and urban furniture creatively as part of their participation in mountain biking.
	Use of the natural landscape	The application of man-made alterations to the natural landscape in order to facilitate particular forms of mountain biking (e.g., the development of rural trail centers and race courses).
Equipment functionality	Purposeful nature to the equipment	The use of equipment as a functional resource in aiding human performance via the identification of key product characteristics (strength, durability, weight and quality) in relation to the purpose that it serves the participant.
	Equipment quality and functionality	The quality of equipment in validating its usefulness in aiding human performance through the association of products with leading professional mountain bikers. This was also achieved through the identification within the adverts of favorable product reviews to develop a sense of product quality.


**Table 2:** The three identity characteristic themes associated with mountain biking that were noted to varying between the various formats of the sport.

Identity Themes	Codes	Description of observations
Sporting characteristics	Celebration of sporting success	The attachment of products to successful athletes (e.g., riders winning world cup races).
	The adoption of a ‘sporting’ visual identity	Use of performance related cycling kit such as lycra or downhill race uniforms to identify riders as competitors.
	Competing and training to compete	The identification of practices performed outside of competition that increases performance abilities for participants (e.g., training, use of training aids and sports science) as well as actually competing in organized and structured events. This also had elements of modern phenomena related to competition such as gamesmanship.
	Professionalization of amateur participants	Reference being made to the psychological aspect of competition to emphasis a professional approach within amateur sports participants. In addition this also included the democratization of access to professional standard equipment for sub-elite riders.
The importance of risk	Risk taking behaviors	Specific examples of risk taking behaviors such as ‘hucking’ in freeriding and the performance of dangerous tricks and stunts. Also the identification of the consequences of risk taking (e.g., serious injury or death).
	The need for protection	The wearing of protective equipment such as knee and elbow pads, neck braces and also the adopted use of full-face helmets.
Activity aesthetics	Demonstration of creativity	The emphasis of artistry and creativity within the activity format through the performance of tricks.


It was noted during the ECA phase of the study that two characteristics appeared commonly across all formats of mountain biking (see **Table 1**). The presence of ‘places of play’ and ‘equipment functionality’ within the sample was noted to emerge so frequently that these were considered to be universal characteristics evident across all formats of mountain biking. However, the three remaining categories (sporting characteristics, the importance of risk, and activity aesthetics) were found to differ between the various styles of mountain biking (see **Table 2**). Therefore, these were carried forward for statistical investigation in the second phase of this research.

In taking this approach this does in no way diminish the importance of places of play and equipment functionality as identity themes in mountain biking as their universality demonstrates them to be key features across the sport as a whole. However, the purpose of this study was to unpick the differences in identity across the various formats of mountain biking and therefore it was the differences noted under semiotic analysis that were explored and evaluated further in the second phase of this research.

## Phase 2: Quantitative Content Analysis (QCA) Method

As has been noted within the initial phase of this research. sporting characteristics, the importance of risk, and activity aesthetics appeared to present areas of difference and could therefore facilitate the production of archetypes of mountain biker identity. It therefore became important to establish if and where differences between these three identity themes occurred. Returning to the sample and using the initial ECA findings, a second stage of data collection and analysis was conducted. This took the form of a QCA using the approach formulated by Berelson (1952) and described by Riff et al. (2014) for advertisement-based research. Individual advertisements were assessed for the presence of each of the seven codes, which emerged from the ECA findings and related three identity themes (risk taking, competitiveness and activity aesthetics). If an individual code was present within the advertisement then it was scored as such. These scores were then accumulated under the three identity themes, meaning that seven codes were looked for in each individual advertisement. The cumulative presence of the codes, under each of the identity themes, led to each advertisement within the sample having three individual scores for competitiveness, risk taking and activity aesthetics. It was these cumulative scores that were used to conduct a statistical examination of this data.

The codes that were sought out within each individual advertisement were as follows:

Competitiveness:

(1) The celebration of sporting success through achievement or the identification of symbols of success (e.g., world championship stripes).
(2) The presence of participants wearing ‘racing kit or racing uniform.’
(3) Competing in events or training for events.
(4) The professionalization of amateur sport through training, psychology or high-tech equipment.

Risk-taking:

(1) Behaviors and practices identified that could result in serious injury or death.
(2) The identification of protective equipment that goes beyond the basics of a standard helmet and gloves (e.g., full face helmet, knee pads or neck brace).

Activity Aesthetics:

(1) The demonstration of creative and artistic practices (e.g., tricks) within the activity format.

In line with the recommendations of Jamieson (2004) and Cohen et al. (2007), non-parametric analysis methods were employed due to the ordinal nature of the data analyzed in this phase of the study. Initial examinations were conducted using a Kruskal–Wallis *H* test (Kruskal and Wallis, 1952) to establish intergroup difference. As there is no *post hoc* method that can be applied to establish where differences exist between groups, a series of Mann–Whitney *U* tests (Mann and Whitney, 1947) were employed, comparing each form of mountain biking in turn with one another. These eight groups were Cross Country, Downhill, Enduro Downhill, Freeride, All Mountain, Dirt Jumping, Street Riding and Trail riding as have been described previously. With this being the case, the Mann–Whitney *U* test (Mann and Whitney, 1947) required seven comparisons to be made and therefore a Bonferroni correction was carried out (*p* = 0.007) to ensure that no type I error was made within the analysis (see Fields, 2013).

The initial findings of this phase indicated similarities in subscale scores between some formats of mountain biking. These were then drawn together into four broader categories, which were then retested again using the method detailed above. These new categories were labeled as Cross County (XC), Downhill (DH), Freestyle (FS) and Trail (TR) and are discussed in the detail in the results section below. However, in retesting these wider grouping categories for the existence of difference only three comparisons were required for the Mann–Whitney *U* tests (Mann and Whitney, 1947). Therefore, a Bonferroni correction was again made to the probability level (*p* = 0.017, see Fields, 2013) for these additional tests of difference.

## Phase 2: QCA Results

Initial examination of the scores for each of the identity themes, across the various formats present within the sample was conducted using a Kruskal–Wallis *H* test (Kruskal and Wallis, 1952). This demonstrated that significant differences between the eight formats of mountain biking did exist for competitiveness (*p <* 0.001), risk taking (*p <* 0.001) and activity aesthetics (*p <* 0.001). Therefore each of these identity characteristics was tested further using a series of Mann–Whitney *U* tests (Mann and Whitney, 1947) to establish where the intergroup differences were apparent between riding styles.

Competitiveness was the factor that most clearly delineated styles of mountain biking (see **Table 3**). Three styles of mountain biking (enduro downhill, downhill and cross country) stood out as showing a significantly greater affinity to competitiveness than other forms of mountain biking. It is notable that no significant differences were found between these three styles of mountain biking when they were tested against one another. Likewise, street riding, trail riding, dirt jumping, all mountain and freeride did not show any significant variation between themselves either, prompting the proposal of two groups of mountain biking; one high competitive cohort and one correspondingly lower in competitiveness.

**Table 3:** Comparative association of competition between riding styles.

	Low competitiveness					High competitiveness		
		
	Street riding	Trail riding	Dirt jumping	All mountain	Freeride	Enduro downhill	Downhill	Cross country
Street riding (*n* = 24)		*P = 0.471 r = -0.06*	*p = 0.696 r = -0.05*	*p = 0.388 r = -0.09*	*p = 0.253 r = -0.12*	***p < 0.001 r = -0.72^∗∗^***	***p < 0.001 r = -0.50^∗∗^***	***p < 0.001 r = -0.55^∗∗^***
Trail riding (*n* = 142)			*p = 0.682 r = -0.03*	*p = 0.734 r = -0.02*	*p = 0.351 r = -0.06*	***P < 0.001 r = -0.65^∗∗^***	***p < 0.001 r = -0.69^∗∗^***	***p < 0.001 r = -0.70^∗∗^***
Dirt jumping (*n* = 48)				*p = 0.539 r = -0.06*	*p = 0.311 r = -0.09*	***p < 0.001 r = -0.70^∗∗^***	***p < 0.001 r = -0.58^∗∗^***	***p < 0.001 r = -0.63^∗∗^***
All mountain (*n* = 71)					*p = 0.620 r = -0.04*	***p < 0.001 r = -0.68^∗∗^***	***p < 0.001 r = -0.63^∗∗^***	***p < 0.001 r = -0.66^∗∗^***
Freeride (*n* = 73)						***P < 0.001 r = -0.64^∗∗^***	***p < 0.001 r = -0.61^∗∗^***	***p = 0.001 r = -0.64^∗∗^***
Enduro downhill (*n* = 27)							*p = 0.247 r = -0.10*	*p = 0.059 r = -0.19*
Downhill (*n* = 106)								*p = 0.162 r = -0.10*
Cross country (*n* = 77)								


*Mountain bike styles are ranked from left to right in order of their mean rank scores for this identity characteristic. Significant differences shown in bold. ^∗^at *p* < 0.007, ^∗∗^at *p*<0.001, with correlations ranked from left to right within the table.*

For risk-taking the distinctions were less obvious. However, it was still possible to discern a high and low category for this identity characteristic (see **Table 4**). Both cross country and trail riding were found to score significantly lower for risk taking than all other formats, and therefore appear to be obvious candidates for a proposed low risk group. However, these can also be joined by all mountain, which was found to be significantly lower in this characteristic than enduro downhill, dirt jumping, street riding, downhill and freeride. It also did not differ significantly when compared to trail riding. Although, it was found to be significantly higher than cross country (*p* = 0.001), which was the lowest scoring group in terms of risk in the sample. The fact that the difference between all mountain and enduro downhill (the next highest risk-taking format) occurred with an above moderate effect size (*r* = -0.38) means that placing all mountain in the low risk taking category and enduro downhill in the higher group for this identity theme was deemed appropriate.

**Table 4:** Comparative association of risk-taking between riding styles.

	Low risk			High risk				
		
	Cross country	Trail riding	All mountain	Enduro downhill	Dirt jumping	Street riding	Downhill	Freeride
Cross Country (*n* = 77)		*p = 0.068 r = -0.12*	***p = 0.001 r = -0.28^∗^***	***p < 0.001 r = -0.66^∗∗^***	***p < 0.001 r = -0.69^∗∗^***	***p < 0.001 r = -0.83^∗∗^***	***p < 0.001 r = -0.73^∗∗^***	***p < 0.001 r = -0.85^∗∗^***
Trail riding (*n* = 142)			*p = 0.010 r = -0.18*	***p < 0.001 r = -0.54^∗∗^***	***p < 0.001 r = -0.63^∗∗^***	***p < 0.001 r = -0.70^∗∗^***	***p < 0.001 r = -0.72^∗∗^***	***p < 0.001 r = -0.84^∗^***
All mountain (*n* = 71)				***p < 0.001 r = -0.38^∗∗^***	***p < 0.001 r = -0.47^∗∗^***	***p < 0.001 r = -0.56^∗∗^***	***p < 0.001 r = -0.57^∗∗^***	***p < 0.001 r = -0.73^∗∗^***
Enduro downhill (*n* = 27)					*p = -0.502 r = -0.08*	*p = 0.167 r = -0.19*	*p = 0.045 r = -0.17*	***p < -0.001 r = -0.44^∗∗^***
Dirt jumping (*n* = 48)						*p = 0.392 r = -0.10*	*p = 0.132 r = -0.12*	***p < -0.001 r = -0.40^∗∗^***
Street riding (*n* = 24)							*p = 0.797 r = -0.02*	***p < 0.001 r = -0.36^∗∗^***
Downhill (*n* = 106)								***p < 0.001 r = -0.38^∗∗^***
Freeride (*n* = 73)								


*Mountain bike styles are ranked from left to right in order of their mean rank scores for this identity characteristic. Significant differences shown in bold. ^∗^ at *p* < 0.007, ^∗∗^ at *p* < 0.001, with correlations ranked from left to right within the table.*

Although all mountain is described here as belonging in a low risk-taking category, it would arguably appear at the upper reaches of this group and the hierarchy developed here should be viewed as a continuum rather than truly discrete categories. A similar situation was also seen in the high-risk category, where freeride displayed a significantly stronger attachment to risk-taking identities than all other formats of mountain biking and therefore the conclusion can be drawn that within the group most associated with risk-taking, freeride is the category that sits at the head of the spectrum.

The statistics displayed in **Table 5** show that freeride, dirt jumping and street riding, while not differing from each other were found to significantly attach more strongly to activity aesthetics and thus form the group that presented this identity more strongly. While the other formats (trail riding, cross country, enduro downhill and downhill) become the alternative low aesthetics group it must be noted that there were significant differences between several of these styles of mountain biking (trail riding and all mountain; trail riding and downhill; and cross country and downhill), however these were found with low effect sizes (*r* = -0.14, *r* = 0.24, *r* = -0.20 respectively*).* By comparison the highest format in the low aesthetics group (downhill) compared to the lowest in the high category (freeride) was found to be significant with a much greater effect size (*r = -*0.45), adding weight to the proposed categorization of styles of mountain biking.

**Table 5:** Comparative association of activity aesthetics between riding styles.

	Low Aesthetic Association					High Aesthetic Association		
		
	Trail Riding	Cross Country	All Mountain	Enduro Downhill	Downhill	Freeride	Dirt jumping	Street riding
Trail riding (*n* = 142)		*p* = *1 r* = *-0.00*	*p* = *0.045 r* = *-0.14*	***p = 0.001 r = -0.25^∗^***	***p < 0.001 r = -0.24^∗∗^***	***P < 0.001 r = -0.62^∗∗^***	***p < 0.001 r = -0.70^∗∗^***	***p < 0.001 r = -0.85^∗∗^***
Cross country (*n* = 77)			*p = 0.139 r = -0.12*	*p = 0.016 r = -0.24*	***p = 0.006 r = -0.20^∗^***	***p < 0.001 r = -0.58^∗∗^***	***p < 0.001 r = -0.66^∗∗^***	***p < 0.001 r = -0.83^∗∗^***
All mountain (*n* = 71)				*p = 0.307 r = -0.10*	*p = 0.087 r = -0.13*	***p < 0.001 r = -0.53^∗∗^***	***p < 0.001 r = -0.61^∗∗^***	***p < 0.001 r = -0.77^∗∗^***
Enduro downhill (*n* = 27)					*p = 0.744 r = -0.03*	***p < 0.001 r = -0.38^∗∗^***	***p < 0.001 r = -0.48^∗∗^***	***p < 0.001 r = -0.68^∗∗^***
Downhill (*n* = 106)						***p < 0.001 r = -0.45^∗∗^***	***p < 0.001 r = -0.51^∗∗^***	***p < 0.001 r = -0.62^∗∗^***
Freeride (*n* = 73)							*p = 0.457 r = -0.0.7*	*P = 0.029 r = -0.22*
Dirt jumping (*n* = 48)								*p = 0.124 r = -0.18*
Street riding (*n* = 24)								


*Mountain bike styles are ranked from left to right in order of their mean rank scores for this identity characteristic. Significant differences shown in bold ^∗^ at *p* < 0.007, ^∗∗^ at *p* < 0.001, with correlations ranked from left to right within the table.*

From these findings it is possible to cluster formats of mountain biking into four categories: Cross Country (XC) demonstrates highly competitive but low risk and low aesthetic characteristics; DH, made up of downhill and enduro downhill, demonstrates a highly competitive and risk-orientated nature with low aesthetics; FS, consisting of street riding, dirt jumping and freeride, can be characterized as being low competitive pursuits with strong attachments to risk and aesthetic; and Trail Riding (TR), made up of trail riding and all mountain, is weakly attached to all three characteristics.

Through repeat testing, again using an initial Kruskal–Wallis *H* test (Kruskal and Wallis, 1952), these new groups (DH, XC, FS, and TR) were also found to differ significantly from one another (competitiveness, *p* < 0.001; risk-taking, *p* < 0.001; activity aesthetics, *p* < 0.001). These new groups were then tested using a series of Mann–Whitney *U* tests (Mann and Whitney, 1947) to establish where the intergroup differences existed.

Downhill was found to possess a significantly greater attachment to risk than TR (*p<*0.001, *r = -0*.64) and XC (*p <* 0.001, *r* = -0.64) as did FS (TR- *p <* 0.001, *r* = -0.70; XC- *p <* 0.001, *r = -*0.69). However, FS was found to present a significantly greater risk identity than DH (*p* = 0.002, *r* = -18) but no significant difference was found between XC and TR. Both XC and DH were found to be significantly more competitive than TR (XC- *p* < 0.001, *r* = 0.69; DH- *p* < 0.001, *r* = -0.69) and FS (XC- *p <* 0.001, *r* = -0.69; DH- *p* < 0.001, *r* = -0.66) but not in comparison to each other. For aesthetic, FS scored significantly higher than all other categories (XC- *p* < 0.001, *r* = -0.55; TR- *p* < 0.001, *r* = -0.64; DH- *p* < 0.001, *r* = -0.49) and DH was found to be significantly higher than both XC (*p* = 0.007, *r* = -0.19) and TR (*p* < 0.001, *r* = -0.20). No significant differences in activity aesthetic were found between XC and TR.

**FIGURE 1:**
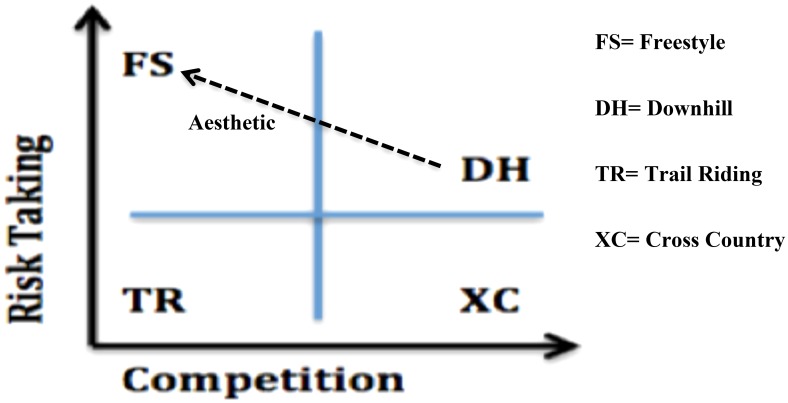
A model of difference between formats of mountain biking.

From the results of the analysis of the regrouped data it is clear that both activity aesthetics and risk taking show a similar trend with both XC and TR being low in these characteristics, FS being the highest and DH being best described as moderate. The performing of tricks and aesthetic movements in mountain biking naturally and quite logically involves taking risks. As an example of this, the main form of aesthetic movement portrayed within the sample was where riders performed tricks in the air while jumping. This obviously creates an additional risk factor and the link between risk taking and aesthetics within the sample of advertisements appeared to occur simultaneously. This possibly indicates that activity aesthetics is simply a form of heightened risk taking rather than a separate identity theme in its own right. If this is the case then the relationship between competitiveness and risk taking becomes the primary focus of identity difference in mountain biking, with activity aesthetic being a factor which differentiates between moderate risk taking from more extreme and dangerous formats of the sport. This relationship can be seen modeled in **Figure 1** and this dynamic became a major focus of the analysis conducted within the third phase of this study.

## Phase 3: Multidimensional Scale Analysis (MDS) Method

McEwan and Weston (2017) analysis of the mountain bike market made use of an MDS analysis to model differing segments. This approach to grouping and classifying results was replicated in a final test conducted on the QCA data. In taking this approach, this study was able to retest and corroborate the groupings found in the initial QCA phase of the study.

The data was analyzed through the use of the mean scores for each of the eight formats of mountain biking in relation to the three previously established identity characteristics (competition, risk taking and aesthetic behavior). MDS analysis allows for the measurement of dichotomous items (see Coleman, 1957; Kruskal and Wish, 1978; DeSarbo et al., 1997; Borg and Groenen, 2005; Mugavin, 2008), in this case forms of mountain biking and each characteristic. Individual styles of mountain biking were compared pair-wise and differences between group mean scores were placed in an upper-triangular matrix ready for analysis using the Proxscal algorithm (Kruskal and Wish, 1978).

An initial principal component analysis (PCA) was conducted, which was followed by the production of a common space diagram again using the Proxscal algorithm (Kruskal and Wish, 1978). The common space diagram was interpreted using the findings of the QCA analysis and also through direct comparison with the results of McEwan and Weston (2017) analysis of the mountain bike market.

## Phase 3: MDS Analysis Results

**FIGURE 2:**
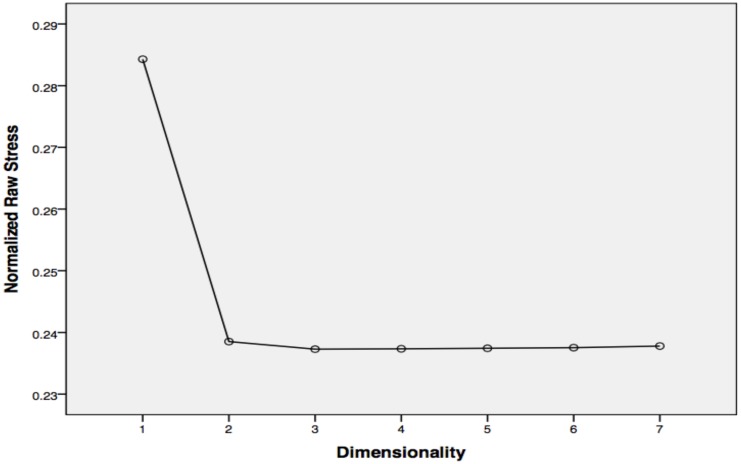
MDS scree plot showing two-dimensional solution.

The PCA (see screen chart in **Figure 2**) shows the elbow point in the distribution of raw stress scores occurring at two dimensions, indicating that there are two rather than three differences between styles of mountain biking. This corresponds to the suggestion drawn from the QCA results that indicate activity aesthetic to be an extension of risk taking rather than an identity characteristic in its own right. A bi-dimensional common space diagram was therefore produced, again using the Proxscal algorithm (Kruskal and Wish, 1978), in order to map the proximities of mountain bike styles based on the QCA results found within this research.

**FIGURE 3:**
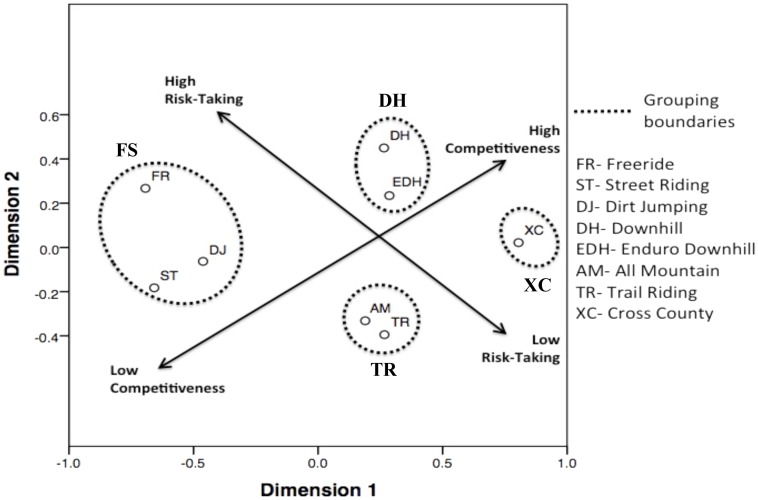
Interpreted bi-dimensional MDS common space diagram showing the clustering of riding styles into the four groupings of Freestyle (FS), Downhill (DH), Cross Country (XC) and Trail Riding (TR).

As shown in **Figure 3** this data was then interpreted in two ways. Firstly, the groupings that were found through the QCA analysis were replicated in the proximities shown on the common space diagram, providing further weight to the categories that have been proposed. **Figure 3** also shows the two key identity differences (risk taking and competitiveness) as axes superimposed on the common space chart, again reinforcing the initial QCA findings.

The QCA analysis found that FS was significantly higher in activity aesthetic and thus represents increased risk-based practices and this, in connection with the MDS analysis, can be seen represented on the MDS common space diagram (see **Figure 3**) and corresponds with the previously proposed model of the relationship between differences in identity across the four categories of mountain biking, as shown in **Figure 1**.

In comparison to McEwan and Weston (2017) study of the mountain bike market, the MDS common space results found within this study show notably similar proximities between the differing styles of mountain biking. However, it also needs to be stated that the interpretation of the results in this study leads to a difference in the clustering of mountain bike styles. Where McEwan and Weston (2017) found six categories of mountain biking based on products and the participant opinions, thus allowing for the establishment of distinct market segments (cross country, trail riding, all mountain, enduro downhill, gravity and freestyle), this study found that, based on identity, mountain biking consisted of four categories of riding formats (FS, DH, XC, and TR).

## Discussion

In developing a model of the pluralized mountain biking market, McEwan and Weston (2017) concluded by posing a question regarding how this could possibly be reflected in participant identities. This study attempted to address this question by evaluating the identity characteristics of each format of mountain biking through a semiotic analysis of mountain biking magazine advertisements. The findings of this study allow an insight into the identity narratives at the heart of particular formats of mountain biking and allow for the proposal of a model of participant archetypes that will be explored within the discussion presented below.

Lifestyle sports have been demonstrated to present strong identities that individuals can associate with (Wheaton, 2004a) and by deciding to take part in an extreme sport, participants begin a process of identity negotiation fuelled by the need to demonstrate subcultural authenticity (Beal and Weidman, 2003; Wheaton and Beal, 2003). Therefore, in extreme sports self-narratives become mediatised and marketable as commodities (Bennett and Lachowetz, 2004). In particular, in the sport of mountain biking, the work of Huybers-Withers and Livingston (2010) highlights how the media can be used to create and promote an identity in an extreme sport. However, Huybers-Withers and Livingston (2010) conducted their analysis based solely on the gendered nature of the identities associated within mountain biking. Although undoubtedly relevant to the analysis presented here, their work does not significantly account for the other factors of identity differences within the sport and therefore the result of this study provide a further and differing perspective on identity within the pluralized sport of mountain biking.

The results of this study provide evidence to suggest that extreme sports identities are not homogenous and that viewing all formats of a sport like mountain biking as being similar is overly simplistic. The results of this study support the notion that mountain biking is pluralized, not only in its markets (see McEwan and Weston, 2017) but also within the identities bound up within differing formats of the sport.

### Mountain Biking Identities

Although five identity characteristics were found to be present within the sample of advertisements, it was notable that two emerged as being common across all forms of mountain biking. The results highlight links to a strong reliance upon technology and the man-made nature of the environments in which mountain biking takes place across all forms of mountain biking. This provides a common ground for all mountain biking formats and highlights the fact that although differences in identity have been found through this research, it is also recognized that similarities also exist. However, the purpose of this study was to establish areas of divergent identity and therefore the discussion presented here will center on the differentiating factors found within the results.

Three identity characteristics emerged from this study that could provide a level of differentiation between the various styles of mountain biking. The emergence of risk taking is a factor that has commonly been associated with extreme sports (Wheaton, 2004a; Rinehart, 2007; Zuckerman, 2007; Barlow et al., 2013, 2015; Frühauf et al., 2017) and indeed the term extreme is itself designed to place an emphasis on the risks involved. Equally Humphreys (2003) highlights the impact of the new leisure movement on the development of aestheticism and artistry within extreme sport, and again this emerged within this research. However, extreme sports have been previously described as possessing “counter cultural philosophies” (Donnelly, 1988, p. 74) that challenge the traditional sports construct (Maguire, 1999) and reject conventional competition (Beal, 1995; Humphreys, 2003; Wheaton, 2003; Wheaton and Beal, 2003). Therefore, it is notable that many traditional sporting characteristics were found within the semiotic analysis of the sample of advertisements, countering the popular image of extreme sports. This provides an example of the varying association of mountain biking formats to each of the three identity characteristics initially established within this study justified the need to further and deeper analysis this area.

### Competitiveness

Sport is best defined from a sociological perspective using the framework devised by Guttmann (1978) in his ground-breaking work, *From Ritual to Records.* Invoking the Freudian concepts of mastery (Freud, 1924), he was able to delineate constructed activities (sports) from simple acts of play and define sport as being an organized and governed contest between co-facilitatory opponents. However, in extreme sports that contain no rules or have no externalized competition, then this thoroughly modern, rather than postmodern definition becomes problematic. Repeated reference is made within the extreme sport literature to participants rejecting traditional forms of competition (see Midol, 1993; Beal, 1995; Wheaton, 2004a, 2007, 2013; Donnelly, 2006). What is clear from the findings of this research is that certain mountain biking formats (cross country, downhill and enduro downhill) do conform to the framework set out by Guttmann (1978). The obvious point here is that the association to, or the rejection of, competitiveness is not universal, highlighting the non-homogenous nature of mountain biking identities.

If an activity conforms to the traditional construct of the ‘contract to contest’ between opponents, under the rules and regulations of a controlling authority, then it must be considered a sport under Guttmann (1978) definition. Equally if these mountain biking formats (cross country, downhill and enduro downhill) are to be categorized as sports then can they simultaneously remain extreme sports? Where other styles of mountain biking fall outside of this definition, the appearance is that mountain biking is made up of some formats, which are traditional sporting activities, while others portray the accepted narrative of the rejection of competition and are therefore extreme sports.

In its early development, mountain biking went through a process of sportification (Savre et al., 2010) but later developments (e.g., freeride) reject the competitive identity. The findings within this study provide evidence that suggests that a more complex competitive/non-competitive dynamic is present within this emerging sport than had previously been observed. Whilst this is an area that clearly requires further analysis, the present results demonstrate how difficult it is to fully categorize extreme sports using older models as a point of definition. It also highlights the need to revise the current thinking on what makes a sport a sport, without having to refer simply to competition.

Where competitiveness was noted within advertisements there were examples of imagery that would not have been out of place if connected to modern sport. The use of race kit serves to set competitors apart and create a sporting visual identity, thus reinforcing the competitive self-narrative. This outwardly displays symbolic sporting commitment that conforms to ideals of dramaturgy (Goffman, 1959). By contrast, other forms of mountain biking displayed a visual persona designed to convey a relaxed attitude that creates a contesting identity narrative to that of competitive formats. Where racing is portrayed as being serious and requiring of commitment, non-racing styles of mountain biking are depicted as being laid-back and relaxed. Symbols of identity such as sports kit can be of use in equal measure to bring individuals together while also allowing groups to distinguish themselves from one another (Eitzen, 2012, p. 42). On the one hand, a distinction between competitors and non-competitors can be framed around the wearing of race kit, but equally the type of race clothing worn denotes the style of mountain biking an individual is competing in. Downhill racers were observed wearing moto-cross inspired clothing, while enduro downhillers wore baggy shorts and t-shirts. Cross country racers were adorned in lycra which would not have been out of place on road cyclists. So visual identity presents a significant characteristic for individuals engaged in competitive mountain biking.

There were other obvious symbols of competition (e.g., race numbers, racing images, celebration of sporting success, etc.) within race orientated mountain biking formats. However, there was also significant weight given to training in order to compete. This again is a coherent narrative that exists within traditional sports where the focus is on training to achieve. The ideal of achievement was widespread across all competitive formats of mountain biking and the use of heroes (professional riders) to emphasize the importance of success was clear. Hofstede et al. (1990) highlight this within their model of manifestations of culture, where heroes embody the attributes that are esteemed within the culture and thus represent its values. In the case of competitive mountain biking, this means sporting success and professional riders play a functional role in representing the subcultural values that reinforce this identity characteristic.

In reviewing the element of competitiveness, the conclusion can be drawn that a sporting identity in the traditional sense explored by Guttmann (1978) exists within some formats of mountain biking. McEwan (2016, p. 277) highlighted the role of mastery in his discussion of mountain bike participants’ characteristics and the results established within this research provide further evidence to corroborate his theory of the emergence of the non-competitive “neo-sportsman.” However, this could equally be the case in a number of postmodern sports and therefore this highlights the need for further analysis of the phenomenon that McEwan (2016) describes and how appears in extreme sports more generally.

### Risk Taking and Activity Aesthetics

If extreme sports participants have in the past been caricatured as risk takers then the result of this study indicate that this is an unsubtle stereotype, which fails to reflect the diversity of identities in a sport such as mountain biking. To a large degree the association to risk suits the narrative of deviance that is prized within extreme sports subcultures. However, the adrenalin junkie myth has already been largely debunked (see Brymer, 2010). Undoubtedly, lifestyle sports contain elements of risk, which in some cases can be extreme in nature (Palmer, 2002, 2004; Simon, 2002). However, it has also been suggested that the risk itself is not the main attraction for participants in extreme sports (Brymer and Oades, 2008; Brymer, 2010). This is complicated by the fact that participants in extreme sports have been shown to possess higher sensation seeking traits than non-extreme sports participants (Cronin, 1991; Campbell et al., 1993; Jack and Ronan, 1998; Diehm and Armatas, 2004; Gomà-i-Freixanet, 2004; Gomà-i-Freixanet et al., 2012) and therefore if experiencing danger is not the main driver of participation, the sensation it provides is still significant (Zuckerman, 2007). However, even this notion has now been contested, with recent research beginning to take into account factors such as emotion regulation and agency within the risk sports experience (see Barlow et al., 2013).

The key observations made within this study connected to risk were twofold but both serve the purpose of reinforcing a sensation seeking and risk-based identity. On the one hand there is the promotion of risk-based practices, such as performing tricks or ‘hucking’ (performing large drops on a mountain bike). It was notable that within advertisements where tricks were being performed, that risk was a strongly associated element of the behavior. The insinuation within risk orientated advertisements was clear in demonstrating that under certain formats of mountain biking, risk taking is an inherently ingrained identity characteristic. This arguably provides the perception that in order to establish group membership and authenticity (Beal and Weidman, 2003; Wheaton and Beal, 2003) it becomes nearly compulsory for participants to engage in risk based subcultural behaviors.

Risk and danger obviously have possible consequences and this was demonstrated in several example advertisements, which included images of injury and crashes. To a great extent this serves a functional purpose, in that it develops and reinforces a strong core identity narrative for participants to benchmark themselves against as members of a subcultural community. Not only do participants have to take risks but they also have to be cognizant of the consequences (serious injury or death) should their actions go wrong. This symbolically differentiates between high and low risk forms of mountain biking and also extends to visual representation through the prevalence of protective equipment. Mountain bikers wear protective equipment regardless of the format they participate in (e.g., helmet and gloves) but in downhill and freeride there were strong connections to the use of more specialist protection (e.g., body armor, full-face helmets and neck braces). These obviously serve a functional purpose but significantly, they also provide a visually symbolic impression to outsiders, reinforcing the activity itself as being high risk and requiring extra security in the form of bodily protection and further reinforcing a risk taking identity.

Initial analysis indicated that activity aesthetic was a characteristic involved in mountain biking identity. However, further evaluation made it clear that rather than it being an independent factor, it was a constituent part of risk-taking. As previously noted, all aesthetic behavior present within advertisements contained a strong element of risk. The analysis demonstrated that the formats of mountain biking that are more closely associated to extreme high risk are the aesthetic or freestyle formats. This aesthetic risk taking arguably could represent a substitution of one form of masculinity for another. As has been shown in other contexts, masculinity in extreme sports can take many guises (Beal, 1996; Robinson, 2004; Wheaton, 2004b) and in this instance competition and mastery of others appears to have been replaced by risk taking connected to aesthetics, particularly in freestyle mountain biking. However, this is not a firm conclusion of this study and further academic investigation of this area is strongly suggested.

### Modeling Identity Archetypes in Mountain Biking

**FIGURE 4:**
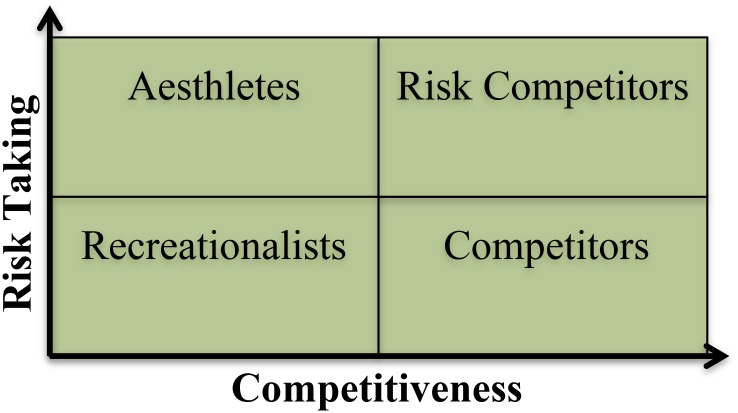
Proposed model of mountain biking identity archetypes.

The results of the study presented here made it possible to draw together forms of mountain biking to create four rationally categorized groups. McEwan and Weston (2017) established six mountain bike markets. However, when identity rather than consumer products are observed it is clear that four clusters of riding styles emerge. The impact of developing these four format groups for mountain biking, along with the identification of risk and competition as defining identity characteristics, meant that it was possible to classify the structural relationship between the different styles of the activity. Based on this, four archetypal identities have been created, formulated around competitiveness and risk taking (which also includes aesthetics, see **Figure 4**). Each archetype represents participants from categorized clusters of riding styles. Recreationalists represent trail riding and all mountain, Competitors denotes cross country, Risk Competitors embody downhill and enduro downhill racing and finally, Aesthletes represent the freestyle forms of mountain biking (street riding, dirt jumping, and freeride).

Competitors are athletes (amateur or professional) who engage in low risk formats of mountain biking but compete regularly and train to prepare for events. Similarly, Risk Competitors also compete and train for events but differ from Competitors in the risk-based component of their style of riding. Aesthlete as they have been referred to here, are extreme high-risk takers that do not to associate with competition. The final category of the Recreationalists can best be described as what they are not. Recreationalist do not engage in competition and are low risk takers in comparison to both Aesthletes and Risk Competitors but share a pattern of low risk taking with the Competitors.

It is not possible to couch the findings of this study concerning risk within the context of previous research on mountain biking specifically as no studies beyond those centring on the epidemiology of injury have been conducted thus far. However, in previous research, extreme sports have more broadly been described as being high risk (Wheaton, 2004a; Donnelly, 2006; Rinehart, 2007). In addition, they have also been described as rejecting competition (see Beal, 1995; Wheaton and Beal, 2003; Wheaton, 2004a, 2007, 2013; Donnelly, 2006). When these two elements are combined and compared to the four archetypes developed within this research, the Aesthletes fit this stereotype most convincingly. However, it is important to note that this research has also identified three other archetypes and these do not readily conform to this depiction of extreme sports. Therefore this questions this description of the nature of extreme sports and the depiction of the risk taking anti-competitor within a homogenous sense would appear to be overly simplistic based on the findings within this study.

Building on previous research on the mountain bike market (see McEwan and Weston, 2017) this study moved the discourse into an analysis of identity. The findings of this study show competitiveness and risk taking to be component identity characteristics in mountain biking. It is recognized that the identity characteristics found in this study are based on the representation made within advertisements and that although this represents an idealized norm it does not automatically follow that these characteristics will be present amongst mountain bike participants themselves. This leads to possible future analysis of these factors as personality traits building on previous research in this area (e.g., Cazenave et al., 2007; Castanier et al., 2010), thus giving this area of study an additional psychological focus. Further research in this field of mountain biking would serve to link the visual identities found within this study with possible trait characteristics of riders themselves. Conducting future research in the area of personality traits would serve to confirm if the advertising materials within the mountain bike market are correctly aligned to the personality traits of the consumers. This study found strong divergences between identities in differing formats of the sport and it may be that these materials could be targeted better with an understanding of how personality traits connect with the identity characteristics found within this study. In practical terms, this information along with the findings of this study would be of use to those promoting products, services and facilities within the mountain bike market.

## Conclusion

The findings of this research represent a contribution to knowledge in this field of study via the establishment of the identity archetypes in the sports of mountain biking, which further the findings of McEwan and Weston (2017), which relate to the differing markets within the sport. The study findings presented here suggest that differing styles of mountain biking can be categorized under one of four groups (FS, XC, TR, and DH) and that participants themselves can be broadly defined under one of four archetypes (Competitors, Risk Competitors, Recreationalists and Aesthletes). The study found evidence highlighting two differing identity narratives that signify the differences between participant archetypes (competitiveness and risk taking). Initial analysis suggested that aesthetic was a third differentiating characteristic, however this was established as a component of extreme risk taking. Indeed it was found that the category with the highest association with risk taking (freestyle) was also the category with the strongest link to aesthetic. It was therefore concluded that within the sport of mountain biking the twin processes of competitiveness and risk taking act as defining identity characteristics and that these would provide a fruitful source for future research and analysis. In particular, a focus on the psychological trait characteristics (linked to competitiveness and risk taking) of those taking part in mountain biking would further illuminate this area of study and serve to further the conclusions within this research. Equally, it is noted that the analysis presented here focuses on a defined set of idealized identities which were drawn from a sample of advertisements for mountain biking products. It must be recognized that the self-identities that riders construct around their participation within the sport of mountain biking may not fully correspond with those found within this study. Therefore, it is recommended that further research is conducted focused specifically on mountain bike participants and their perceptions of the varying identities associated with the sport.

## Author Contributions

KM was responsible for the initial conceptualization of the study and the development of the research design, data collection, data analysis and interpretation, and drafting of the manuscript. NW and PG assisted in the data analysis and interpretation. They also worked on critically refining the manuscript for important intellectual content.

## Conflict of Interest Statement

The authors declare that the research was conducted in the absence of any commercial or financial relationships that could be construed as a potential conflict of interest.
